# Probiotics for glycemic control in patients with type 2 diabetes mellitus: protocol for a systematic review

**DOI:** 10.1186/s13643-019-1145-y

**Published:** 2019-09-03

**Authors:** Thanitsara Rittiphairoj, Krit Pongpirul, Noel T. Mueller, Tianjing Li

**Affiliations:** 1 Department of Epidemiology, Johns Hopkins Bloomberg School of Public Health, Baltimore, MD 21205 USA; 2 Department of Preventive and Social Medicine, Faculty of Medicine, Chulalongkorn University, 1873 Rama IV Road, Pathumwan, Bangkok, 10330 Thailand; 3 Department of International Health, Johns Hopkins Bloomberg School of Public Health, Baltimore, MD 21205 USA; 4 Welch Center for Prevention, Epidemiology and Clinical Research, Baltimore, MD 21205 USA

**Keywords:** Glycemic control, Probiotics, Type 2 diabetes, Systematic review

## Abstract

**Background:**

Type 2 diabetes mellitus (T2DM) is a major public health problem worldwide. It is characterized by the increased concentration of glucose in the blood and leads to damage of the body system, especially blood vessels and nerves. Lifestyle modification is often combined with anti-diabetic therapy as the standard of care for T2DM to maintain the proper blood glucose and to prevent long-term diabetic complications. The role of probiotics in improving glycemic control has been investigated in several randomized controlled trials (RCTs). Previous systematic reviews and meta-analyses, including different sets of trials have concluded an overall beneficial effect of probiotics in patients with T2DM. At least two RCTs with a longer treatment duration have been published since the publication of existing reviews.

**Methods:**

We will conduct a systematic review of RCTs that evaluated the effectiveness and safety of probiotics for glycemic control in T2DM patients. Primary outcomes are fasting blood glucose and glycosylated hemoglobin (A1c). Secondary outcomes are plasma insulin, blood lipid profile, adverse events, and cost associated with the intervention and hospital visits. We will search PubMed, Embase, and the Cochrane Central Register of Controlled Trials (CENTRAL) in *The Cochrane Library*, and trial registries. Two reviewers will independently screen titles and abstracts, review full texts, extract information, and assess the risk of bias. We will summarize the results both qualitatively and statistically. We will use random-effects model for meta-analysis.

**Discussion:**

This systematic review aims to examine whether probiotics are effective and safe for glycemic control in T2DM patients. Evidence generated from this review will inform clinical and public health practice and future research.

**Systematic review registration:**

CRD42019121682

**Electronic supplementary material:**

The online version of this article (10.1186/s13643-019-1145-y) contains supplementary material, which is available to authorized users.

## Background

### Description of the condition

Diabetes mellitus is a chronic disease characterized by impaired insulin sensitivity or production, which leads to increased blood glucose concentration and eventually damage to the body system, especially blood vessels and nerves [1]. Type 2 diabetes mellitus (T2DM) is the most common form of diabetes [2]. The standard treatment of T2DM is lifestyle modification, often combined with anti-diabetic therapy (oral anti-diabetic medication with or without insulin therapy) to maintain the proper blood glucose and to prevent long-term diabetic complications [3].

Patients with poorly controlled blood glucose are at risk for both microvascular complications such as renal, retinal, and neuropathy diseases, as well as macrovascular complications such as peripheral vascular diseases and coronary diseases. These complications lead to morbidity and mortality [2, 4, 5].

Diabetes is a major public health problem. In 2017, it was estimated that 451 million people have diabetes worldwide. The prevalence of diabetes is anticipated to increase to 693 million by 2045 [6]. In the United States (US), diabetes was the 7th major cause of death in 2015 [7]. Of the 7.2 million patients with a diabetes diagnosis in 2014 in the US, 1.5 million patients also had major cardiovascular diseases such as coronary diseases and strokes, and 108,000 patients had lower-extremity amputations [7]. In 2017, average health care spending for diabetic patients was USD 16,750 per year, which were 2.3 times higher than health spending for non-diabetic patients [8].

### Description of the intervention

Probiotics are live bacteria and yeasts that may benefit health [9, 10]. Probiotics exist in fermented foods and beverages (e.g. yogurt, milk, cheeses, kimchi) and in functional foods (e.g. soy-based products, cabbage, maize). Probiotics are also found in dietary supplements, in the form of tablets, capsules, powders, and liquid extracts [10–12]. The two strains used widely in functional foods and dietary supplements are *Lactobacillus* and *Bifidobacterium* [13]. Historically, these two aerobic strains have been easiest to culture; new strains, even anaerobic strains, are now being increasingly studied.

Probiotics work by changing the composition of the gut microbiome, in theory helping to achieve microbial balance. Some probiotics purport to increase intestinal motility, improve intestinal barrier function, stimulate immune response, and modulate inflammatory gene expression in the gut [10, 14–17]. Evidence from clinical trials suggests that probiotics have a beneficial effect for managing gastrointestinal diseases such as irritable bowel syndrome [18], diarrhea [19], and non-gastrointestinal diseases such as allergic diseases [20] and genitourinary infections in women [21].

### Mechanisms through which probiotics may improve glucose homeostasis

The change in the gut microbiome and its fermentation have been associated with T2DM [22, 23]. It is postulated that the overgrowth of some gram-negative bacteria may influence risk of T2DM through inflammatory pathways. For example, excessive gram-negative bacterial fragment lipopolysaccharide (LPS) may lead to a leakage of gut barrier and, as a result, chronic systemic inflammation [24, 25]. The gut microbiota may also influence glucose metabolism by modulating the glucagon-like peptide-1 (GLP-1), one of enteroendocrine peptides produced by L-cell in the gut. The secretion of GLP-1 is associated with a reduction in gastric emptying time and food intake, and an increase in insulin secretion [26, 27].

### Why it is important to do this review

The role of probiotics in improving glycemic control has been investigated in several randomized controlled trials (RCTs). While some trials found that probiotics could lower the blood sugar and decrease insulin resistance [28–33], the evidence is inconsistent [34–37]. Previous systematic reviews and meta-analyses have concluded an overall beneficial effect of probiotics in patients with T2DM. However, the literature searches in these systematic reviews do not seem to be comprehensive and the trials included all had a short treatment duration and follow-up period [38–43]. Since the publication of these systematic reviews, at least two RCTs with a longer treatment duration have been published [32, 33].

## Objective

To assess the effectiveness and safety of probiotics for glycemic control in patients with T2DM through a systematic review.

## Methods

We have registered the systematic review with PROSPERO registration number CRD42019121682 and have followed the Preferred Reporting Items for Systematic Review and Meta-Analysis Protocols (PRISMA-P) 2015 statement [44]. We used PRISMA 2015 checklist to ensure the quality of the protocol (see Additional file 1).

### Criteria for considering studies for this review

#### Types of studies

We will include only RCTs. We will include reports of RCTs irrespective of their publication status and language.

#### Type of participants

We will include RCTs of participants of 18 years or older, of any sex, race/ethnicity, and diagnosed with prediabetes (diagnosis as defined by the individual trial) or T2DM (diagnosis as defined by the individual trial). We will accept RCTs in which participants had any duration and severity of the disease and were treated with any anti-diabetic therapy. We will exclude trials of patients with type 1 diabetes mellitus or gestational diabetes because of different disease pathways and mechanisms.

#### Type of interventions

We will include RCTs that the interventions are probiotics or synbiotics, which are defined as probiotics plus prebiotics (non-digestible food ingredients) [17], of any type (i.e. fermented foods, functional foods, and dietary supplements) administered by any route with or without the combination of standard treatment as defined by trialists. Standard treatment for T2DM includes lifestyle modification combined with anti-diabetic therapy (oral anti-diabetic medication with or without insulin therapy [3]. The comparison intervention will be placebo, prebiotic (for synbiotic trials), or standard treatment alone (as defined by trialist). We will exclude trials in which the dose of probiotics (in the specific metric as colony-forming unit [CFU]) was not clearly specified.

#### Type of outcome measures

Most trials on this topic had a short-term duration of probiotics treatment (shorter than 12 weeks) [28, 30, 31, 37]. However, some believed that a long-term probiotics consumption is needed for understanding its effect [32, 33]. Therefore, we will examine each outcome described below at two time points: short term (shorter than 12 weeks) and long term (greater than or equal to 12 weeks). Within each timeframe, we will choose the outcome measurement at the longest follow-up time point. For example, if a trial reported results at both 4 and 8 weeks, we will analyze the result at 8 weeks for the short-term outcome.

#### Primary outcomes

- Mean change in fasting blood glucose (mg/dL) from the baseline;
- Mean change in glycosylated hemoglobin (%) from the baseline.

#### Secondary outcomes

- Mean change in plasma insulin (μU/ml) from the baseline;
- Mean change in triglyceride, cholesterol, low-density lipoprotein (LDL), and high-density lipoprotein (HDL) (mg/dL) from the baseline.

#### Adverse outcomes

- Proportion of participants experienced probiotics related adverse events such as abdominal cramping, abdominal pain, nausea, taste disturbance, soft stools, diarrhea, flatulence, bloating, and systemic infection such as septicemia and endocarditis [45].

#### Health services outcomes

- Costs associated with the intervention;
- Mean number of hospital or health professional visits.

### Search methods for identification of studies

#### Electronics searches

We will work with an information specialist for designing a search strategy, which will use both medical subject headings and keywords. We will search MEDLINE via PubMed, Embase, and the Cochrane Central Register of Controlled Trials (CENTRAL) in *The Cochrane Library*. We will search clinical trials registries for ongoing and recently completed trials via clinicaltrials.gov and World Health Organization International Trials Registry and Platform (www.whoint/ictrp/search/en/ISRCTN;,Registry). We will not apply language or date restrictions. See Additional file 2 for details of search strategies for each database.

#### Searching other resources

We will search references cited in included trials. We will also search the website of the manufacturers of probiotics for information regarding additional unpublished or forthcoming trials.

### Data collection and analysis

#### Selection of studies

We will use Covidence to manage all citations identified from the search [46]. After removing duplicates from the search results, two review authors will work independently to screen the titles and abstracts. We will classify each record as relevant or non-relevant for full-text review. Two review authors will independently review full-text reports of trials classified as relevant from the title and abstract screening to determine the final eligibility. For reports that are excluded at the full-text screening stage, we will document the reason(s) for exclusion. We will generate a study flow diagram that describes the identification of trials. At each stage of the screening process, we will resolve disagreements through discussion.

#### Data extraction and management

We will use an electronic data collection system (e.g. Covidence, Systematic Review Data Repository (SRDR), Qualtrics) to manage data extraction. We will design a data extraction form and refine it by pilot testing. Two review authors will independently extract the following data items: (1) general information, including trial name and registration information; (2) trial characteristic, including trial design, location, setting, and inclusion/exclusion criteria; (3) characteristic of participants, including age, sex, race/ethnicity, severity of the diabetes, and comorbidities; (4) details of interventions, including type, strain, composition of probiotics, dose, duration of treatment, co-interventions (anti-diabetic standard therapy); (5) details of comparison interventions; (6) outcomes as described under “type of outcome measure” section.

We will resolve data extraction discrepancies through discussion. We will contact the trial authors for incomplete or unclear information. If the trial authors do not respond for 14 days, we will pursue analyses using available data.

#### Assessment of risk of bias in included studies

Two authors will work independently to assess the risk of bias in the included trials using the Cochrane Risk of Bias tool 2.0 [47]. We will assess each of the following domains:

- Bias arising from the randomization process;
- Bias due to deviations from intended intervention;
- Bias due to missing outcome data;
- Bias in measurement of the outcome;
- Bias in selection of the reported result.

We will assign each domain as low, high, and unclear risk of bias. We will contact the trial author if there is not enough information to assess. If the trial authors do not respond for 14 days, we will pursue assessment using available data. We will resolve the disagreement through discussion. We will present our risk of bias assessment in the “Risk of bias” summary tables.

#### Assessment of reporting bias

We will search for trial protocols and trial registration information. We will compare the outcomes and analyses specified in these records with those reported in the journal articles. Reporting bias is suspected when there was a change in primary or secondary outcomes, or analysis plan.

#### Measure of treatment effect

For continuous data, we will present results as mean difference with 95% confidence intervals (CIs). For dichotomous data, we will present results as risk ratio with 95% CIs.

#### Assessment of heterogeneity

We will assess clinical and methodological heterogeneity by examining participant characteristics, probiotics type, duration of probiotics usage and dose, outcomes, and the study of design. We will assess statistical heterogeneity using the *I*^2^ and *χ*^*2*^ statistics. *I*^2^ statistic of 0 to 40% might not be important; 30 to 60% may represent moderate heterogeneity; 50 to 90% may represent substantial heterogeneity; 75 to 100% considerable heterogeneity [48]. For *χ*^*2*^ test, we will assess the included trials for statistical heterogeneity with a *P* value of less than 0.10 (statistically significant).

#### Data synthesis

We will provide qualitative analysis of trials and their results following standard 4.2 that conduct a qualitative synthesis, chapter 4 of *Finding What Works in Health Care: Standards for Systematic Reviews* [49]. If there is no considerable clinical, methodological, and statistical heterogeneity, we will combine the data using a random-effects meta-analysis. We will analyze data using Review Manager version 5.3 [50].

#### Quality of evidence

We plan to use the Grading of Recommendation Assessment, Development and Evaluation (GRADE) approach to assess the quality of evidence for the primary outcomes (i.e., mean change in fasting blood glucose (mg/dL) from the baseline; mean change in glycosylated hemoglobin (%) from the baseline). We will use the five GRADE considerations (i.e., risk of bias, imprecision, inconsistency, indirectness, and publication bias) and grade each outcome as follows [51]:

- High quality defined as we are very confident that the true effect lies close to that of the estimate of the effect.
- Moderate quality defined as we are moderately confident in the effect estimate: the true effect is likely to be close to the estimate of the effect, but there is a possibility that it is substantially different.
- Low quality defined as our confidence in the effect estimate is limited: the true effect may be substantially different from the estimate of the effect.
- Very low quality defined as we have very little confidence in the effect estimate: the true effect is likely to be substantially different from the estimate of effect.

#### Subgroup analysis and investigation of heterogeneity

We will undertake a subgroup analysis by types of probiotics and duration of usage; and by types of co-intervention received.

#### Sensitivity analysis

We will exclude trials at high risk of overall bias to assess the robustness of the results. We will conduct additional sensitivity analyses to determine the impact of any post hoc decisions made during the review process.

## Additional files

Additional file 1: PRISMA-P (Preferred Reporting Items for Systematic review and Meta-analysis Protocols) 2015 checklist. Recommended items to address in a systematic review protocol. (DOCX 79 kb)

Additional file 2: Details of search strategies for each database. (DOCX 23 kb)

## Data Availability

Not applicable at this stage. Data will be available as supplementary files, once the systematic review is completed.

## Abbreviations

CENTRAL: Cochrane Central Register of Controlled Trials
CFU: Colony-forming unit
CIs: Confidence intervals
GRADE: Grading of Recommendation Assessment, Development and Evaluation
HDL: High-density lipoprotein
LDL: Low-density lipoprotein
LPS: Lipopolysaccharide
PRISMA-P: Preferred Reporting Items for Systematic review and Meta-analysis Protocols
RCTs: Randomized controlled trials
SRDR: Systematic Review Data Repository
T2DM: Type 2 diabetes mellitus
US: United States
