# Supplementary material for: Probiotics for glycemic control in patients with type 2 diabetes mellitus: protocol for a systematic review
Source: Syst Rev. 2019 Sep 3;8:227. doi: 10.1186/s13643-019-1145-y (PMC6720889; doi:10.1186/s13643-019-1145-y)
Supplement: Supplementary file 2 — Details of search strategies for each database. (DOCX 23 kb) [file 13643_2019_1145_MOESM2_ESM.docx]

**Additional file 2: Details of search strategies for each database**

**Database: PubMed search strategy**

#1 “Diabetes Mellitus”[Mesh]

#2 “Diet, Diabetic”[Mesh]

#3 “Blood Glucose”[Mesh]

#4 “Glucose Intolerance”[Mesh]

#5 “Insulin Resistance”[Mesh]

#6 “Insulin”[Mesh]

#7 “diabetes”[tw] OR diabetic*[tw] OR “type2DM”[tw] OR “T2DM”[tw] OR prediabetic*[tw]

#8 “blood glucose”[tw] OR “serum glucose”[tw] OR “blood sugar”[tw] OR “glucosaemia”[tw] OR “plasma glucose”[tw] OR “serum sugar”[tw] OR “glucosemia”[tw] OR “glycaemia”[tw] OR “glycemia”[tw]

#9 glucose intoleranc*[tw] OR glucose toleranc*[tw] OR “impaired glucose”[tw]

#10 “insulin*”[tw] OR “iszilin”[tw]

#11 (#1 OR #2 OR #3 OR #4 OR #5 OR #6 OR #7 OR #8 OR #9 OR #10)

#12 “Probiotics”[Mesh]

#13 “Prebiotics”[Mesh]

#14 “Synbiotics”[Mesh]

#15 “Gastrointestinal Microbiome”[Mesh]

#16 “Lactobacillus”[Mesh]

#17 “Bifidobacterium”[Mesh]

#18 probiotic*[tw] OR prebiotic*[tw] OR synbiotic*[tw]

#19 alimentary canal flor*[tw] OR alimentary tract flor*[tw] OR bowel flor*[tw] OR bowel microb*[tw] OR digestive canal flor*[tw] OR digestive tract flor*[tw] OR enteric flor*[tw] OR enteric microb*[tw] OR gastrointestinal microb*[tw] OR gastrointestinal microflora*[tw] OR gastrointestinal flor*[tw] OR gastrointestinal canal flor*[tw] OR gastrointestinal tract flor*[tw] OR gastrointestine flor*[tw] OR gastrointestine tract flor*[tw] OR gut microb*[tw] OR gut microflor*[tw] OR gut bacteria*[tw] OR gut flor*[tw] OR gastric microb*[tw] OR gastric microflor*[tw] OR gastric flor*[tw] OR intestine bacteri*[tw] OR intestine bacteria chang*[tw] OR intestine bacterial flor*[tw] OR intestine microbial flor*[tw] OR intestine microflor*[tw] OR intestinal microb*[tw] OR intestinal microflor*[tw] OR intestinal flor*[tw] OR intestinal bacteri*[tw] OR intestinal bacterial flor*[tw] OR intestinal canal flor*[tw] OR “intestinal microorganism”[tw] OR intestinal tract flor*[tw]

#20 “Lactobacilus”[tw] OR “Lactobacillus acidophilus”[tw] OR “Lactobacillus brevis”[tw] OR “Lactobacillus casei”[tw] OR “Lactobacillus delbrueckii”[tw] OR “Lactobacillus fermentum”[tw] OR “Lactobacillus gasseri”[tw] OR “Lactobacillus helveticus”[tw] OR “Lactobacillus johnsonii”[tw] OR “Lactobacillus reuteri”[tw] OR “Lactobacillus rhamnosus”[tw] OR “Lactobacillus salivarius”[tw] OR “Lactobacillus sporogenes”[tw]

#21 “Bifidobacterium”[tw] OR “Bifidobacterium animalis”[tw] OR “Bifidobacterium bifidum”[tw] OR “Bifidobacterium breve”[tw] OR “Bifidobacterium longum”[tw]

#22 “L. acidophilus”[tw] OR “L. brevis”[tw] OR “L. casei”[tw] OR “L. delbrueckii”[tw] OR “L. fermentum”[tw] OR “L. gasseri”[tw] OR “L. helveticus”[tw] OR “L. johnsonii”[tw] OR “L. reuteri”[tw] OR “L. rhamnosus”[tw] OR “L. salivarius”[tw] OR “L. sporogenes”[tw] OR “B. animalis”[tw] OR “B. bifidum”[tw] OR “B. breve”[tw] OR “B. longum”[tw]

#23 (#12 OR #13 OR #14 OR #15 OR #16 OR #17 OR #18 OR #19 OR #20 OR #21 OR #22)

#24 randomized controlled trial [pt]

#25 controlled clinical trial [pt]

#26 randomized [tiab]

#27 placebo [tiab]

#28 drug therapy [sh]

#29 randomly [tiab]

#30 trial [tiab]

#31 groups [tiab]

#32 (#24 OR #25 OR #26 OR #27 OR #28 OR #29 OR #30 OR #31)

#33 animals [mh] NOT humans [mh]

#34 (#32 NOT #33)

#35 (#11 AND #23 AND #34)

**Database: Embase search strategy**

#1 ‘Diabetes Mellitus’/exp

#2 ‘Diabetic Diet’/exp

#3 ‘Glucose blood level’/exp

#4 ‘Glucose Intolerance’/exp

#5 ‘Insulin Resistance’/exp

#6 ‘Insulin’/exp

#7 (diabetes OR diabetic* OR type2DM OR T2DM OR prediabetic*):ab,ti,de,kw

#8 (‘blood glucose’ OR ‘serum glucose’ OR ‘blood sugar’ OR glucosaemia OR ‘plasma glucose’ OR ‘serum sugar’ OR glucosemia OR glycaemia OR glycemia):ab,ti,de,kw

#9 (‘glucose intoleranc*’ OR ‘glucose toleranc*’ OR ‘impaired glucose’):ab,ti,de,kw

#10 (insulin* OR iszilin):ab,ti,de,kw

#11 (#1 OR #2 OR #3 OR #4 OR #5 OR #6 OR #7 OR #8 OR #9 OR #10)

#12 ‘Probiotic agent’/exp

#13 ‘Prebiotic agent’/exp

#14 ‘Synbiotic agent’/exp

#15 ‘Intestine flora’/exp

#16 ‘Lactobacillus’/exp

#17 ‘Bifidobacterium’/exp

#18 (probiotic* OR prebiotic* OR synbiotic*):ab,ti,de,kw

#19 (‘alimentary canal flor*’ OR ‘alimentary tract flor*’ OR ‘bowel flor*’ OR ‘bowel microb*’ OR ‘digestive canal flor*’ OR ‘digestive tract flor*’ OR ‘enteric flor*’ OR ‘enteric microb*’ OR ‘gastrointestinal microb*’ OR ‘gastrointestinal microflora*’ OR ‘gastrointestinal flor*’ OR ‘gastrointestinal canal flor*’ OR ‘gastrointestinal tract flor*’ OR ‘gastrointestine flor*’ OR ‘gastrointestine tract flor*’ OR ‘gut microb*’ OR ‘gut microflor*’ OR ‘gut bacteria*’ OR ‘gut flor*’ OR ‘gastric microb*’ OR ‘gastric microflor*’ OR ‘gastric flor*’ OR ‘intestine bacteri*’ OR ‘intestine bacteria chang*’ OR ‘intestine bacterial flor*’ OR ‘intestine microbial flor*’ OR ‘intestine microflor*’ OR ‘intestinal microb*’ OR ‘intestinal microflor*’ OR ‘intestinal flor*’ OR ‘intestinal bacteri*’ OR ‘intestinal bacterial flor*’ OR ‘intestinal canal flor*’ OR ‘intestinal microorganism’ OR ‘intestinal tract flor*’):ab,ti,de,kw

#20 (Lactobacilus OR ‘Lactobacillus acidophilus’ OR ‘Lactobacillus brevis’ OR ‘Lactobacillus casei’ OR ‘Lactobacillus delbrueckii’ OR ‘Lactobacillus fermentum’ OR ‘Lactobacillus gasseri’ OR ‘Lactobacillus helveticus’ OR ‘Lactobacillus johnsonii’ OR ‘Lactobacillus reuteri’ OR ‘Lactobacillus rhamnosus’ OR ‘Lactobacillus salivarius’ OR ‘Lactobacillus sporogenes’):ab,ti,de,kw

#21 (Bifidobacterium OR ‘Bifidobacterium animalis’ OR ‘Bifidobacterium bifidum’ OR ‘Bifidobacterium breve’ OR ‘Bifidobacterium longum’):ab,ti,de,kw

#22 (‘L. acidophilus’ OR ‘L. brevis’ OR ‘L. casei’ OR ‘L. delbrueckii’ OR ‘L. fermentum’ OR ‘L. gasseri’ OR ‘L. helveticus’ OR ‘L. johnsonii‘ OR ‘L. reuteri’ OR ‘L. rhamnosus’ OR ‘L. salivarius’ OR ‘L. sporogenes’ OR ‘B. animalis’ OR ‘B. bifidum’ OR ‘B. breve’ OR ‘B. longum’):ab,ti,de,kw

#23 (#12 OR #13 OR #14 OR #15 OR #16 OR #17 OR #18 OR #19 OR #20 OR #21 OR #22)

#24 ‘crossover procedure’:de

#25 ‘double-blind procedure’:de

#26 ‘randomized controlled trial’:de

#27 ‘single-blind procedure’:de

#28 (random* OR factorial* OR crossover* OR cross NEXT/1 over* OR placebo* OR doubl* NEAR/1 blind* OR singl* NEAR/1 blind* OR assign* OR allocat* OR volunteer*):de,ab,ti

#29 (#24 OR #25 OR 26 OR #27 OR #28)

#30 (#11 AND #23 AND #29)

**Database: CENTRAL search strategy**

#1 MeSH descriptor: [Diabetes Mellitus] explode all trees

#2 MeSH descriptor: [Diet, Diabetic] explode all trees

#3 MeSH descriptor: [Blood Glucose] explode all trees

#4 MeSH descriptor: [Glucose Intolerance] explode all trees

#5 MeSH descriptor: [Insulin Resistance] explode all trees

#6 MeSH descriptor: [Insulins] explode all trees

#7 diabetes OR diabetic* OR type2DM OR T2DM OR prediabetic*

#8 blood glucose OR serum glucose OR blood sugar OR glucosaemia OR plasma glucose OR serum sugar OR glucosemia OR glycaemia OR glycemia

#9 glucose intoleranc* OR glucose toleranc* OR impaired glucose

#10 insulin* OR iszilin

#11 MeSH descriptor: [Probiotics] explode all trees

#12 MeSH descriptor: [Prebiotics] explode all trees

#13 MeSH descriptor: [Synbiotics] explode all trees

#14 MeSH descriptor: [Gastrointestinal Microbiome] explode all trees

#15 MeSH descriptor: [Lactobacillus] explode all trees

#16 MeSH descriptor: [Bifidobacterium] explode all trees

#17 probiotic* OR prebiotic* OR synbiotic*

#18 alimentary canal flor* OR alimentary tract flor* OR bowel flor* OR bowel microb* OR digestive canal flor* OR digestive tract flor* OR enteric flor* OR enteric microb* OR gastrointestinal microb* OR gastrointestinal microflora* OR gastrointestinal flor* OR gastrointestinal canal flor* OR gastrointestinal tract flor* OR gastrointestine flor* OR gastrointestine tract flor* OR gut microb* OR gut microflor* OR gut bacteria* OR gut flor* OR gastric microb* OR gastric microflor* OR gastric flor* OR intestine bacteri* OR intestine bacteria chang* OR intestine bacterial flor* OR intestine microbial flor* OR intestine microflor* OR intestinal microb* OR intestinal microflor* OR intestinal flor* OR intestinal bacteri* OR intestinal bacterial flor* OR intestinal canal flor* OR intestinal microorganism OR intestinal tract flor*

#19 Lactobacilus OR Lactobacillus acidophilus OR Lactobacillus brevis OR Lactobacillus casei OR Lactobacillus delbrueckii OR Lactobacillus fermentum OR Lactobacillus gasseri OR Lactobacillus helveticus OR Lactobacillus johnsonii OR Lactobacillus reuteri OR Lactobacillus rhamnosus OR Lactobacillus salivarius OR Lactobacillus sporogenes

#20 Bifidobacterium OR Bifidobacterium animalis OR Bifidobacterium bifidum OR Bifidobacterium breve OR Bifidobacterium longum

#21 L. acidophilus OR L. brevis OR L. casei OR L. delbrueckii OR L. fermentum OR L. gasseri OR L. helveticus OR L. johnsonii OR L. reuteri OR L. rhamnosus OR L. salivarius OR L. sporogenes OR B. animalis OR B. bifidum OR B. breve OR B. longum

#22 (#1 OR #2 OR #3 OR #4 OR #5 OR #6 OR #7 OR #8 OR #9 OR #10)

#23 (#11 OR #12 OR #13 OR #14 OR #15 OR #16 OR #17 OR #18 OR #19 OR #20 OR #21)

#24 (#22 AND #23)

**Database: clinicaltrial.gov search strategy**

(diabetes OR insulin resistance) AND (probiotics OR prebiotics OR synbiotics OR “gastrointestinal microbiome” OR intestine flora OR Lactobacillus OR Bifidobacterium)

**Database: ICTRP search strategy**

diabetes AND probiotics OR diabetes AND prebiotics OR diabetes AND synbiotics OR diabetes AND gastrointestinal microbiome OR diabetes AND intestine flora OR diabetes AND Lactobacillus OR diabetes AND Bifidobacterium OR insulin resistance AND probiotics OR insulin resistance AND prebiotics OR insulin resistance AND synbiotics OR insulin resistance AND gastrointestinal microbiome OR insulin resistance AND intestine flora OR insulin resistance AND Lactobacillus OR insulin resistance AND Bifidobacterium
